# McClelland’s Regional Anatomy

**Published:** 1892-04

**Authors:** 


### Book Notices.

REGIONAL ANATOMY IN ITS RELATION TO MEDICINE AND SURGERY. By George McClellan, M. D., Lecturer on Anatomy at the Pennsylvania School of Anatomy; Professsor of Anatomy in the Pennsylvania Academy of the Fine Arts. Illustrated from photographs taken by the author from his own dissections, and colored by him after Nature. Vol. I. J. B. Lippincott Co., Philadelphia, 1891.

This important work has been looked for by anatomists with considerable curiosity, because so many collections of anatomical plates with descriptive text have been published within recent years. It will perhaps be most frequently compared, at least in America, with the somewhat similar works of Allen and Weisse. These two authors spent much time in making elaborate dissections and in having them copied for illustrating their respective books. In both of them, however, the artistic work was done by artists who made drawings from the dissections of the authors. In the present work the author has not only made the dissections, but has photographed them and colored the photographs as well, so that the entire work, except the printing and lithographing, is the product of his own hand.

The author of Weisse's Human Anatomy spent a number of years in the preparation of dissections, and the drawings made by an accomplished artist from these dissections were excellent; but the camera used by Dr. McClellan naturally gives more absolutely correct representations of nature. The letter-press in connection with Dr. Weisse's illustrations is not very elaborate, and in this respect also Dr. McClellan's book seems more valuable to the practitioner or student who may be called upon to study the anatomy of any particular region.

Dr. Allen, in his Human Anatomy, has given the profession most satisfactory work in what may be called the practical application of anatomy to medicine and surgery, for his elaborate references to anatomical points pertaining to medicine and surgery are most valuable. The plates, however, which are made in the same way as Dr. Weisse's, do not impress the eye as being absolute representations of structures so much as semi-diagrammatic illustrations. These criticisms are not made to disparage the works of the two authors mentioned, but to lay particular stress upon the great fidelity which the colored photographic plates of Dr. McClellan possess. The appearance and coloring are so faithfully exact that it almost seems as if one were looking upon an injected human cadaver lying on a dissecting-room table. The descriptive text is clear and sufficiently comprehensive for the use of those who refer to the volume for the purpose of refreshing the memory on anatomical details. In fact, it is a text-book of regional anatomy illustrated by perfect plates; not a series of plates with descriptive text.

Dr. McClellan, in his preface, speaks of the value of extemporaneous drawings in teaching, and states his belief that they are far better for that purpose than elaborate, previously-prepared pictures. It is very evident, from this and from his other statements, that he must be an excellent teacher for medical students. If, however, the actual cadaver cannot be obtained for the study of anatomy or for the rehearsing of such study, the plates he has here published answer the purpose as perfectly as it is possible for illustrations to do. In his descriptions he uses terms which he says experience has shown him to be more easily understood and remembered by the student than purely technical ones.

Instead of giving a description of each individual member of the osseous and ligamentous systems, followed by a detailed descriptive catalogue of every muscle, nerve, vessel, and organ, the author discusses regions of the body. This is eminently satisfactory if a book on anatomy is consulted by a physician or surgeon when making a diagnosis or studying the steps of a proposed operation. The illustrations of actual operations done upon the cadaver are valuable to surgeons especially, and are rather an unusual addition to a book which purports to be simply a treatise on anatomy. It is a feature, however, to which no one will object.

It is evident that the author has avoided the temptation of making the illustrations diagrammatically clear at the expense of truthfulness to the subject pictured. In the descriptions of the plates will be found a statement of how the dissections were prepared on each of the subjects; and in a few instances where additions have been made to the photographic plates, in order to bring out prominently some special points in anatomy, a statement to that effect is made. This feature of the work makes the reader feel that, where such notes have not been added, he is looking upon a faithful representation of the dissected body, and removes all suspicion that the beauty of these plates is due to artificial aids contributed for the purpose of increasing the artistic value at the expense of fidelity. In plate No. 33 there has apparently been shown a rupture of one of the thyroid vessels causing the pigmented arterial injection to stain the surrounding tissues. This imperfection in the subject is truthfully shown in the tinting added to the photograph. One cannot help being pleased to see this evidence of absolute truthfulness on the part of the delineator.

The reviewer in going over the volume began to make a list of those illustrations which seemed to him particularly satisfactory and valuable, but the list soon became so large that it is impossible to give it in such a review as this. In truth, every picture portrays some important anatomical point or points, and the unusual dissections and regions displayed make very many of the plates of surprising interest.

There are some statements in the text to which exception may perhaps be justly taken; for example, on page 2, "the bones of the *head*" is apparently an inaccurate statement for the "bones of the *cranium;*" and on another page the greater relative amount of splintering of the inner table of the cranial bones in cases of fracture of the cranium is erroneously attributed to its vitreous character.

The interest which at the present day is attached to cerebral surgery is a sufficient reason for repeating the following statement of Dr. McClennan: "Repeated examinations of the relations of the fissures to carefully mapped-out points after removal of a disk of bone on the heads of many cadavers have shown the author the fallacy of depending solely upon measurements, and the importance of making the artificial opening in the skull large enough to enable the operator to see the parts exposed." Occasionally the diction is a little obscure, as in the description on page 198 of the action of the sterno-mastoid muscle in causing wry-neck.

The necessities of binding have, unfortunately, obliged the publisher to distribute the plates somewhat unequally throughout the volume. This, as must happen in such cases, separates the text from the plates to which it refers. This is also due in part to the thorough manner in which the author describes the various regions, a method which requires frequent cross references to plates widely separated in the paging.

The book will be received with the greatest pleasure by those who are interested in studying or teaching anatomy, and the publication of the second volume will be awaited with impatience. It is unnecessary to congratulate the author upon the production of this work, because he who gives the amount of labor required for the accomplishment of such a self-imposed task must feel intense gratification at what he has lived to accomplish; and the man that can produce such a book certainly has the taste, judgment, and ability to appreciate its artistic and anatomical worth. J. B. R.

---

PRACTICAL INTESTINAL SURGERY. By F. B. Robinson, M. D. Physician's Leisure Library Series; pp. 200; price, 25 cents. Address: Geo. S. Davis, Publisher, Detroit, Michigan.

---

THE VEST-POCKET ANATOMIST, (founded upon "Gray"). 198 illustrations; 14th revised edition. By C. Henri Leonard, A. M., M. D. Published by the Illustrated Medical Journal Co., Detroit, Mechigan: 1891.

